# miR-23a, miR-146a and miR-301a confer predisposition to Vogt-Koyanagi-Harada syndrome but not to Behcet’s disease

**DOI:** 10.1038/srep20057

**Published:** 2016-01-28

**Authors:** Shengping Hou, Zi Ye, Dan Liao, Lin Bai, Yunjia Liu, Jun Zhang, Aize Kijlstra, Peizeng Yang

**Affiliations:** 1 The First Affiliated Hospital of Chongqing Medical University, Chongqing, China; 2 Chongqing Eye Institute and Chongqing Key Laboratory of Ophthalmology, Chongqing, China; 3 University Eye Clinic Maastricht, Maastricht, The Netherlands

## Abstract

Ninety-eight miRNAs are involved in the immune response. However, the genetic roles of these miRNAs remain unclear in Behcet’s disease (BD) and Vogt-Koyanagi-Harada (VKH) syndrome. This study aimed to explore the association and functional roles of copy number variants (CNV) in several miRNAs with BD and VKH syndrome. Genotyping of CNVs was examined by TaqMan PCR. The expression of miR-23a, transfection efficiency and cytokine production were measured by real-time PCR, flow cytometry or ELISA. First, replication and combined studies for miR-23a, miR-146a and miR-301a demonstrated a similar association with VKH syndrome (Combined: P = 5.53 × 10^−8^; P = 8.43 × 10^−31^; P = 9.23 × 10^−8^, respectively). No association of CNVs of the above mentioned miRNAs was observed in BD patients. mRNA expression of miR-23a showed a positive association with its copy numbers. Additionally, individuals with high copy number of miR-23a show an increased production of interleukin-6 (IL-6), but not IL-8 and monocyte chemoattractant protein-1 (MCP-1) by stimulated PBMCs. miR-23a transfected ARPE-19 cells modulated the production of IL-6 and IL-8, but not MCP-1. Our results suggest that CNVs of miR-146a, miR-23a and miR-301a confer susceptibility to VKH syndrome, but not to BD. The contribution of miR-23a to VKH syndrome may be mediated by increasing the production of IL-6.

Vogt-Koyanagi-Harada (VKH) syndrome and Behcet’s disease (BD) are two commonly seen uveitis entities in China^1^,^2^,^3^. VKH syndrome is a multisystemic autoimmune disorder characterized by bilateral granulomatous panuveitis frequently associated with systemic involvement including vitiligo, poliosis, alopecia, auditory and central nervous system signs^4^. BD is recognized as a systemic inflammatory disorder characterized by non-granulomatous uveitis, recurrent oral, genital ulcers, and skin lesions^5^. Although the exact pathogenesis of these diseases remains unclear, a complex genetic background together with an autoimmune process which is directed against autoantigens or external antigens has been accepted to be the hallmark of both diseases^6^,^7^. Genetic factors are of vital importance in the complex immune process leading to BD and VKH syndrome^8^,^9^. Various studies have identified Single nucleotide polymorphisms (SNPs) in *HLA-B51, IL23R, STAT4, SUMO4, CCR1*, and copy number variants (CNV) at *C4, IL17F, IL23A* to be associated with BD^10^,^11^,^12^,^13^,^14^,^15^,^16^,^17^,^18^,^19^. *HLA-DRB1*/*DQA1, IL23R*-*C1orf141* and *ADO*-*ZNF365*-*EGR2* were considered as genetic factors for the susceptibility to VKH syndrome^20^. The different risk genes combined with different clinical findings of these two uveitis entities suggest that the development of uveitis may be caused by different pathogenetic pathways.

Previous studies showed that miR-146a and pre-miRNA-196a2 genetic variants were associated with BD but not with VKH syndrome^8^,^21^. Additionally, a decreased miRNA-155 expression was found in patients with BD but not in VKH syndrome^22^. These studies suggest that miRNAs may provide clues to explain the different pathogenetic pathways leading to either BD or VKH syndrome.

miRNAs, one of the non-coding RNAs families, have been shown to play a critical role in mediating post-transcriptional regulation of gene expression^23^. As powerful regulators of numerous genes and pathways in immune responses, miRNAs can affect inflammatory and immune mediated diseases via regulating their cellular and molecular targets^24^. Recent studies have found that polymorphisms of miRNAs may lead to autoimmune or inflammatory diseases^25^,^26^. miR-146a, which was related to inflammation and apoptosis processes, has been widely confirmed to be associated with immune diseases such as rheumatoid arthritis (RA), systemic lupus erythematosus (SLE) and psoriasis^27^,^28^,^29^. miR-9 can post-transcriptionally regulate proinflammatory cytokines in human CD4^+^ T cells, such as IL-2^30^, and the miR-23a cluster was shown to repress B-cell development *in vivo*^31^. miR-22, miR-143, miR-205,miR-132 and miR-301a also have been recognized to play important roles in immune functions and autoimmunity^32^,^33^,^34^,^35^,^36^.

Copy number variations (CNVs) belong to structural variations of DNA, and capture more genetic information than SNPs (5%–10% vs <1%). CNVs of various genes are linked to gene expression^37^ and are associated with uveitis^14^,^38^,^39^. However, to our knowledge no reports have appeared concerning miRNAs CNVs and uveitis. We therefore performed a study to examine whether the CNVs in miRNAs were possibly implicated in the pathogenesis of uveitis. Ninety-eight miRNAs are implicated in immune response (Supplementary Table 1), however, only eight immune related miRNAs including miR-146a, miR-23a, miR-22, miR-143, miR-205, miR-9, miR-132 and miR-301a known to have CNVs in the human genome variants database were addressed in the present study. Our results showed that copy number variants of miR-146a, miR-23a and miR-301a are significantly associated with VKH syndrome, but not with BD. Functional studies showed that miR-23a may be implicated in the development of VKH syndrome by the upregulated production of inflammatory cytokines such as IL-6.

## Results

### Clinical feature of patients with BD and VKH syndrome

The clinical characteristics of the uveitis patients were assessed at the time of diagnosis and summarized in Table 1.

### First stage study for copy numbers of investigated miRNAs between patients and controls

A total of 383 VKH syndrome patients, 377 BD patients and 660 healthy controls were enrolled in the first stage study. The most common counts of miRNA gene copy numbers are 2 copies, so comparison between patients and controls was performed after a division into three groups: less than 2, equal to 2 or more than 2 gene copy numbers. The frequencies of copy numbers of the investigated miRNAs (miR-9-3, miR-22, miR-23a, miR-143, miR-146a, miR-205, miR-301a) was not different in BD patients and healthy controls (Table 2). Increased frequencies of more than 2 copies for miR-23a and miR-146a were found in VKH syndrome patients as compared with normal controls (miR-23a > 2, P = 2.00 × 10^−4^, OR = 2.9; miR-146a > 2, P = 1.21 × 10^−11^, OR = 51.98, respectively) (Table 2). An increased frequency of less than 2 copies for miR-301a was found in VKH patients (P = 4.71 × 10^−4^, OR = 3.8) (Table 2).There was no significant difference for the remaining miRNAs including miR-9-3, miR-22, miR-143 and miR-205 copy numbers between VKH patients and healthy controls (Table 2). Two different probes were used to test miR-132 CNVs, however, the result was unreliable and data for miR-132 were excluded from the study.

### The replication and combining studies for copy numbers of miR-23a, miR-146a and miR-301a in VKH patients

To further confirm the results of the first stage study, an additional set of 860 VKH patients and 1631 healthy controls were evaluated for miR-23a, miR-146a and miR-301a copy numbers. The replication study demonstrated that high copy numbers (>2) of miR-23a, miR-146a and low copy number (<2) of miR-301a were consistently linked to the susceptibility to VKH syndrome (miR-23a > 2, P = 5.83 × 10^−5^, OR = 2.11; miR-146a > 2, P = 1.08 × 10^−22^, OR = 4.81; miR-301a < 2, P = 3.87 × 10^−5^, OR = 2.6, respectively) (Table 2). Combining the data from both studies showed that individuals carrying high copy numbers of miR-23a, miR-146a or a low copy number of miR-301a revealed an increased predisposition to VKH syndrome (miR-23a > 2, P = 5.53 × 10^−8^, OR = 2.32; miR-146a > 2, P = 8.43 × 10^−31^, OR = 5.63; miR-301a < 2, P = 9.23 × 10^−8^, OR = 2.9, respectively) (Table 2).

### The expression of miR-23a in individuals with different copy numbers

**Figure 1:**
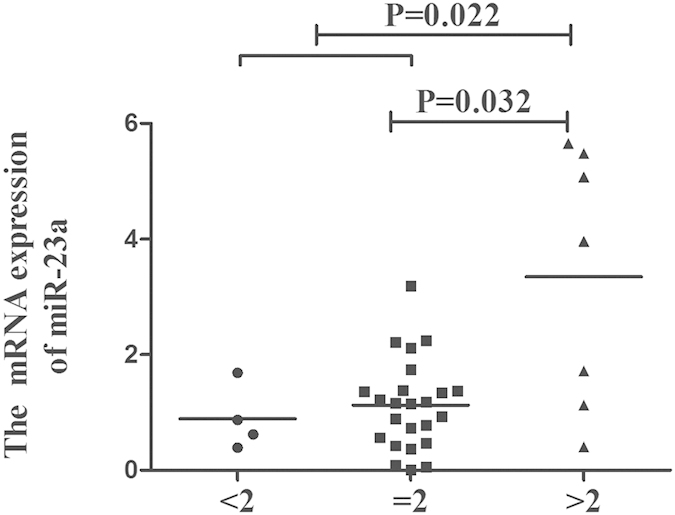
The influence of CNVs in miR-23a on the mRNA levels. The mRNA expression of miR-23a in PBMCs from controls carrying different gene copies of miR-23a (miR-23a < 2: n = 4, miR-23a = 2: n = 24, miR-23a > 2: n = 7). Significance was examined using SPSS’s two independent samples Nonparametric test.

We also tested the relationship between miR-23a and its mRNA expression. A significantly increased mRNA expression of miR-23a was found in the high copy group (miR-23a > 2) as compared with the low copy number group (miR-23a ≤ 2) (miR-23a > 2 vs miR-23a ≤ 2: P = 0.022; miR-23a > 2 vs miR-23a < 2: P = 0.032) (Fig. 1).

### The influence of miR-23a copy number variants on cytokine production in peripheral blood mononuclear cells (PBMCs)

**Figure 2:**
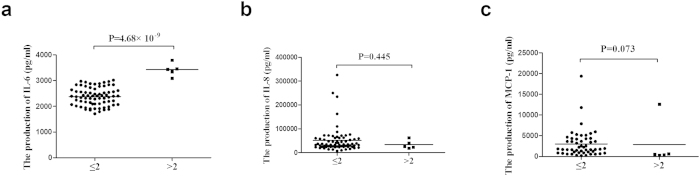
The influence of miR-23a CNVs on cytokines production. The production of IL-6 (**a**), IL-8 (**b**) and MCP-1 (**c**) by stimulated PBMCs from healthy controls carrying different gene copies of miR-23a (miR-23a < 2: n = 72, miR-23a > 2: n = 5). Significance was analyzed by Student’s t test or two independent samples Nonparametric test.

Since miR-23a CNVs correlate with the expression of miR-23a at the transcriptional level, further experiments were performed to investigate whether CNVs of miR-23a affected cytokine production. Seventy-seven normal controls, which are different from those used for the expression study, were enrolled in the examination of cytokine production. As no individual with less than 2 copies was found in the enrolled samples in this study, only groups with more than 2 or equal to 2 gene copy numbers were examined. Previous studies demonstrated that the expression of IL-6, IL-8 and MCP-1 were significantly increased in the vitreous fluid, aqueous humor or serum of uveitis patients^40^,^41^,^42^. Additionally, the treatment with anti-IL-6 antibody could inhibit the severity of a mouse model of uveitis^43^. These three cytokines were therefore examined in this study. An increased production of IL-6 by stimulated PBMCs was found in individuals with a high copy number of miR-23a (P = 4.68 × 10^−9^) (Fig. 2). There was no significant association between the production of IL-8, MCP-1 by stimulated PBMCs and CNV of miR-23a (Fig. 2).

### Optimal concentration of miR-23a mimics and inhibitors in human retinal pigment epithelial cell line (ARPE-19)

**Figure 3:**
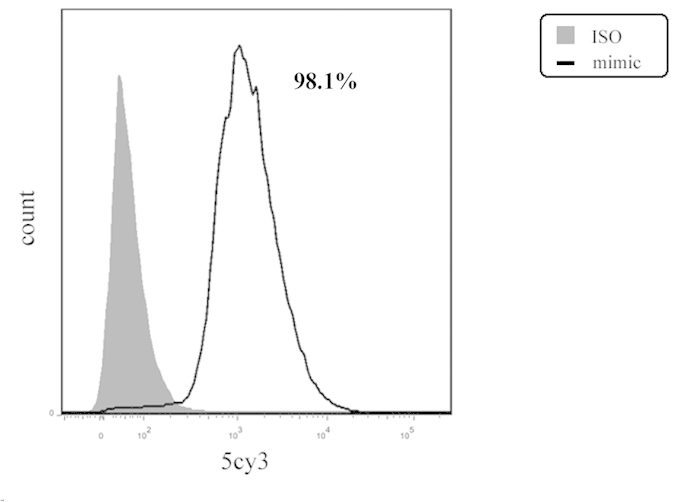
Transfection efficiency of miR-23a mimics and inhibitors in human RPE cells. Results of flow cytometry showed a successful transfection efficiency of miR-23a mimics and inhibitors in human RPE cells, which was up to 98.1% in the experimental group and as little as 0.9% in the control group.

**Figure 4:**
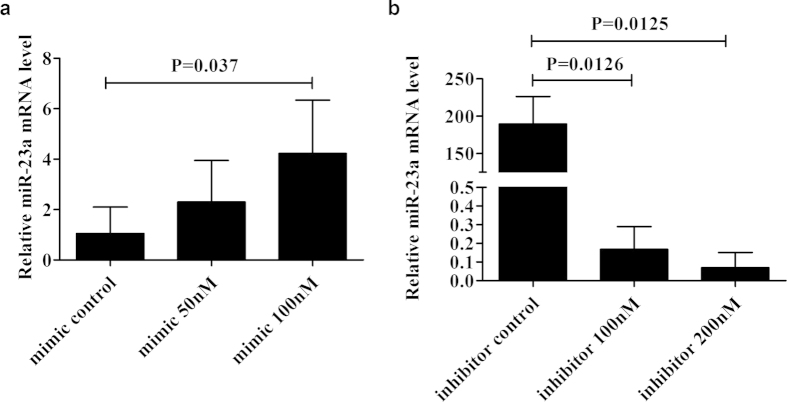
Design condition of miR-23a mimics and inhibitors in human RPE cells. Measurement of miR-23a mRNA expression was performed after 24-hour transfection. For miR-23a mimics, concentration of 100 nM had a stronger over expression effect than 50 nM. For miR-23a inhibitors, concentration of 200 nM had a stronger inhibitory effect than 100 nM. Data was shown as the mean ± SD of three independent experiments. Paired-sample t test was used for statistical analyses.

Results of flow cytometry showed a successful transfection efficiency of 5Cy3-labeled miRNA mimic control in human RPE cells, which was up to 98.1% (Fig. 3). According to the manufacturer’s reagent supplies manual, we chose concentrations of 50 nM and 100 nM for miR-23a mimics and 100 nM and 200 nM for miR-23a inhibitors. To obtain the optimal concentration for transfection, expression of miR-23a was detected by quantitative RT-PCR (qRT-PCR) and compared between different groups. ARPE-19 cells transfected with miRNA mimics at a final concentration of 100 nM showed an optimal overexpression of miR-23a (Fig. 4). For miR-23a inhibitors, concentration of 200 nM had a stronger inhibitory effect than 100 nM. Therefore, retinal pigment epithelium (RPE) cells were transfected with miRNA mimics and inhibitors at a final concentration of 100 nM and 200 nM respectively (Fig. 4).

### miR-23a promotes the expression of IL-6 and IL-8 in human ARPE-19 cells

**Figure 5:**
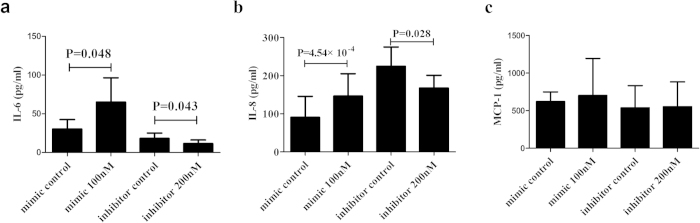
The influence of miR-23a mimics and inhibitors in human RPE cells on cytokine production. RPE cells were transfected with miRNA mimics and inhibitors at a final concentration of 100 nM and 200 nM respectively. After 24 hours, supernatants of transfected RPE cells were collected and measured for the production of IL-6 (**a**), IL-8 (**b**) and MCP-1(**c**) by ELISA (n = 5). Data are shown as mean ± SD. Significance was analyzed by paired samples test.

To elucidate the role of miR-23a in regulating the cytokine production of IL-6, IL-8 and MCP-1 in human RPE cells, cells were transfected with miR-23a mimics as well as inhibitors to affect the endogenous miR-23a level. Results of ELISA showed an increased IL-6 and IL-8 production, but not for MCP-1 in supernatants of RPE cells transfected with miR-23a mimics (P < 0.001) (Fig. 5). Additionally, transfected RPE cells with miR-23a inhibitors showed downregulated expression of IL-6 and IL-8, but not MCP-1 (Fig. 5).

## Discussion

The present study aimed to explore the association of CNVs of eight miRNAs with VKH syndrome and Behcet’s disease. The results showed that three miRNAs including miR-23a, miR-146a and miR-301a are associated with VKH syndrome but not with BD. No association was found between the remaining CNVs in four miRNAs including miR-9-3, miR-22, miR-143, miR-205 and VKH syndrome or BD. The functional study showed that mRNA expression of miR-23a was positively related with its copy numbers. Additionally, increased production of IL-6, but not IL-8 and MCP-1 by stimulated PBMCs was found in the group with a high copy number of miR-23a. Consistently with the result in PBMCs, miR-23a mimics significantly increased the production of IL-6 and IL-8, but not MCP-1 in RPE cells, whereas miR-23a inhibitors suppressed cytokines such as IL-6 and IL-8.

Many miRNAs have been shown to be involved in the development of immune diseases such as multiple sclerosis (MS), rheumatoid arthritis and uveitis^22^,^35^,^44^,^45^. However, the genetic role of these miRNAs in uveitis has to our knowledge not yet been addressed and was therefore the purpose of the study described here. The tested candidate miRNAs were principally chosen due to their role in modulating the immune response. Many immune-related miRNAs have been described. However, only eight miRNAs including miR-146a, miR-23a, miR-22, miR-143, miR-205, miR-9, miR-132 and miR-301ashow gene copy number variation and probes are available to test this.

To the best of our knowledge, this is the first study to identify an association of miRNAs CNVs with human disease. The results showed that high copy numbers of miR-146a, miR-23a and low copy number of miR-301a confer susceptibility to VKH syndrome. Inconsistent with the results in VKH syndrome, no association of these three miRNAs CNVs were found for BD. The discrepancy in the association between VKH syndrome and BD may be partially due to the fact that these three miRNAs including miR-146a, miR-23a and miR-301a may be involved in the development of autoimmune disease rather than inflammatory disease. VKH syndrome is generally considered as autoimmune disease directed against melanocytes, whereas BD is now defined as an autoinflammatory disease^46^,^47^,^48^. miR-23a plays a suppressive role on CD8^+^ T cell effector functions^49^, suggesting the involvement of this miRNA in immune disease. The level of miR-23a was decreased in serum of MS patients^44^, and its rs3745453 variant was associated with susceptibility for MS^50^. Inconsistent with the aforementioned result, this miRNA was found to be upregulated in gastric cancer^51^, suggesting a variable disease involvement for miR-23a. miR-146a acts as a negative regulator of immune cell activation^52^ by repressing its target genes including TRAF6 and IRAK1. A promoter SNP of miR-146a confers risk for SLE by modulating its expression^53^. Zhou *et al*.^8^ also showed the association of a miR-146a SNP with Behcet’s disease, but not with VKH syndrome. However, there are no reports on the association of miR-146a CNV with human disease. Our present study showed that miR-146a CNV may contribute to the risk for human diseases such as VKH syndrome but not BD. These results suggest that certain genetic variants of miR-146a such as gene polymorphisms and copy number variants can both be involved in the development of uveitis.

As the frequency of less than 2 copies of miR-301a is as low as 1.7% in controls, only one sample with less than 2 copies of miR-301a was found in our population of healthy controls. The relationship between the expression of miR-301a and its copy numbers was therefore not examined in this study. Additionally, our group tested the functional role of a genetic variant of miR-146a in uveitis in a previous study^8^. We therefore focused on the functional role between miR-23a CNV and its expression and the effect of miR-23a CNV on the production of inflammatory cytokines. Since there is a marked heterogeneity in the clinical presentation of VKH syndrome and in view of the general treatment regimen of these patients that includes immunosuppressive drugs, we performed functional studies using PBMCs from genotyped normal controls. A positive association was found between miR-23a CNV and its expression in PBMCs. Individuals with a high copy number of miR-23a showed an increased production of IL-6 in PBMCs. Additionally, since the RPE plays an important role in the pathogenesis of uveitis, we also examined the functional role of miRNA in these cells. **S**imilar to the results obtained in PBMCs, we found enhanced productions of IL-6 and IL-8 in transfected RPE cells with miR-23a mimics, whereas IL-6 and IL-8 production was suppressed in RPE cells that were transfected with miR-23a inhibitors. Our results are in agreement with a recent study that showed that miR-23a increased the expression of IL-6 in LPS-stimulated macrophages^54^. Contrary to the aforementioned findings, previous studies showed no significant effect of miR-23a on IL-6 levels in trophoblast cells^55^ and a suppressive role of miR-23a on IL-6 production by purified CD8^+^ T cells^56^. These studies suggest that the role of miR-23a in disease may affect different pathways which may depend on the cell type investigated such as CD8^+^ T cells, PBMCs, RPE cells or macrophages. A previous study also showed that miR-23a has a suppressive role on the production of interferon-γ (IFN-γ)^49^. We tested the expression of IFN-γ by RPE cells but the expression level was below the detection limit of our assay.

It is worthwhile to point out that our experiments concerning miR-23a CNV on cytokine production are unbalanced for the different CNV groups, which may affect the interpretation of these functional studies. Our cDNA bank of healthy controls is however much smaller than our DNA bank and since the frequency of the miR-23a > 2 individuals is relatively small. We were only able to enroll 5 controls for this group in our expression study. Further studies using larger samples are need to further examine the functional role of miR-23a.

In conclusion, we found that high copy numbers of miR-146a, miR-23a and a low copy number of miR-301a confer risk for VKH syndrome, but not for BD. miR-23a may be involved in the development of VKH syndrome by increasing the production of inflammatory cytokines such as IL-6.

## Materials and Methods

### Study population

Individuals selected for this study included a total of 377 BD patients, 1,243 VKH patients and 2,291 healthy controls. All individuals were Han Chinese and were recruited via the First Affiliated Hospital of Chongqing Medical University (Chongqing, China) or the Zhongshan Ophthalmic Center, Sun Yat-sen University (Guangzhou, China). The revised diagnostic criteria 2001 for VKH syndrome and the International Study Group for BD were used to make the diagnosis of VKH syndrome and BD, respectively^57^,^58^. Patients with a doubtful diagnosis were excluded from the study. The healthy controls were age-, and ethnicity-matched with the patients as well as free of any intraocular or systemic inflammatory condition. Written informed consent was obtained from every participant. The present study was approved by the Ethics Committee of the First Affiliated Hospital of Chongqing Medical University (Permit Number: 2009-201008) and adhered to the tenets of the Declaration of Helsinki. This study was registered in the Chinese Clinical Trial Registry (Registration number: ChiCTR-CCC-12002184, Registration time: 2012-05-23).

### DNA extraction and genotyping

Genomic DNA was extracted from peripheral blood by using the QIAamp DNA Blood Mini Kit (QIAGEN,Valencia, CA). Copy numbers of examined genes were determined by the TaqMan-based qPCR which was performed in 96-well optical plates on a 7500 real time PCR system (Applied Biosystems). Nine TaqMan probes (labeled with FAM) specific for our studied genes were as follows: miR-9, Hs05330221_-_cn; miR-22, Hs01095539_-_cn; miR-23a, Hs04021842_-_cn; miR-143, Hs03578207_-_cn; miR-146a, Hs06722002_-_cn; miR-205, Hs07483345_-_cn; miR-301a, CXN1ENO; miR-132, Hs03965136_cn or CCN1FDA. RNaseP (labeled with VIC) was used as the control assay for normalization. Both TaqMan probes and RNaseP were obtained from Applied Biosystems (ABI, FosterCity, CA). Standard thermal cycler conditions were set as follows: 95 °C for10 min, 40 cycles of 95 °C for 15s and 60 °C for 1 min. Measurements of CNVs for each sample were performed in three replicates.

### Cell isolation and culture

PBMCs were obtained from heparinized blood using Ficoll-Hypaque density-gradient centrifugation. Isolated PBMCs were suspended in complete RPMI medium containing 10% fetal calf serum, 2mML-glutamine and 100 U/ml penicillin/streptomycin (Invitrogen, Carlsbad, CA) and were adjusted to 1 × 10^6^ cell/ml. PBMCs were incubated in 24-well plates induced by LPS (100 ng/ml, Sigma, Missouri, USA) at 37 °C for 24 h to detect IL-6, IL-8 and MCP-1.

### Transfection of miRNA mimics and inhibitors in RPE cells

ARPE-19 cells were obtained from the American Type Culture Collection (ATCC, Manassas, VA), and cells at passages 21 to 25 were used for the present experiment. ARPE-19 cells were cultured in DMEM/F12 medium (Invitrogen, Carlsbad, CA) supplemented with 10% FBS, 100 U/ml penicillin and 100 ug/ml streptomycin in an incubator at 37 °C in 5% CO_2_. ARPE-19 cells were seeded in 12-well plates at 5 × 10^5^ cells/well and cultured for 24 h to reach 50–70% confluence and then transfected with miR-23a mimics or inhibitors (RIBOBIO, Guangzhou, China) using Lipofectamine RNAiMAX (Invitrogen) and OPTI-MEM I reduced serum medium (Invitrogen) according to the manufacturer’s protocol for 24 h. The cells were harvested for RNA analyses. Negative control mimics or inhibitors were used as matched controls.

To detect the effect of miR-23a on cytokine production by ARPE-19 cells, the cells were transfected with miR-23a mimics or inhibitors for 6 h and then stimulated with LPS (1 ug/ml, Sigma-Aldrich) for 18 h. The culture supernatants were collected for detection of IL-6, IL-8 and MCP-1 by ELISA.

### Flow cytometry

To evaluate the transfection efficiency of miRNAs in human RPE cells, cells were gently washed with PBS after transfection with 5Cy3 labeled mimics for 24 h and directly analyzed by flow cytometry. Flow cytometry was performed on a FACS Aria cytometer (BD Bioscience, San Diego, CA) and the data were processed using FlowJo software (Treestar, Inc., San Carlos, CA).

### RNA extraction and qRT-PCR

Total RNA, including miRNA, was extracted from unstimulated PBMCs and transfected RPE cells using TRIzol reagent (Invitrogen, USA). RNA concentration was assessed by NanoDrop 2000 (Wilmington, DE). The levels of miRNAs were measured by qRT-PCR using miDETECT A Track™ miRNA qRT-PCR Kit (RiboBio, Guangzhou, China) and performed on ABI 7500 System (Applied Biosystems). The primers for miR-23a, miR-301a and U6 small nuclear RNA were obtained from RiboBio Company (Guangzhou, China). The sequences are covered by a patent. Analyses of miRNA expression were normalized to the expression of internal control U6 using the 2^−ΔΔCT^ method.

### Measurement of cytokines by ELISA

Supernatants of stimulated PBMCs and transfected RPE cells were stored at −80 °C until cytokine measurement. The concentration of IL-6, IL-8 and MCP-1 in the supernatants was measured with a human Duoset ELISA development kit (R&D Systems, Minneapolis, MN) according to the manufacturer’s protocols.

### Statistical analysis

The differences in copy numbers of miRNAs between patients and controls were analyzed by chi-square test using SPSS (v. 17.0; SPSS Inc., Chicago, IL). Bonferroni correction was applied to correct for multiple comparisons. The expression of miRNAs and various cytokines was analyzed by independent samples *t* test or two independent samples Nonparametric test using SPSS 17.0 software. Corrected P values less than 0.05 were considered significant.

## Additional Information

**How to cite this article**: Hou, S. *et al*. miR-23a, miR-146a and miR-301a confer predisposition to Vogt-Koyanagi-Harada syndrome but not to Behcet's disease. *Sci. Rep.* **6**, 20057; doi: 10.1038/srep20057 (2016).

## Supplementary Material

Supplementary Information

## Figures and Tables

**Table 1:** Clinical features of patients and controls enrolled in the present study.

**Clinical features**	**Total**	**%**
**VKH syndrome**	1243	
Mean age (years ± SD)	39.0 ± 13.8	
Male	685	55.1
Female	558	44.9
Uveitis	1243	100
Headache	530	42.6
Scalp allergy	191	15.4
Tinnitus	554	44.6
Dysacusia	406	32.7
Alopecia	476	38.3
Poliosis	447	36.0
Vitiligo	217	17.5
**Behcet’s disease**	377	
Mean age (years ± SD)	33.1 ± 8.3	
Male	329	
Female	48	
Uveitis	377	100
Oral ulcer	377	100
Skin lesions	268	71.1
Genital ulcer	198	52.5
Arthritis	61	16.2
Positive pathergy test	78	20.7
**Controls**	2291	
Mean age (years ± SD)	39.5 ± 10.9	
Male	1291	56.4
Female	1000	43.6

**Table 2:** The association of Copy number variants of miR-9-3, miR-22, miR-23a, miR-143, miR-146a, miR-205 and miR-301a with Behcet’s disease and VKH syndrome.

**Genes**	**Stage**	**CNVs**	**VKH**(frequency)	**BD**(frequency)	**Controls**(frequency)	**P(VKH)**	**OR(95% CI)**	**P (BD)**	**OR(95% CI)**
miR-22	Stage 1	<2	5(1.3%)	12(3.2%)	7(1.1%)	0.721	1.2(0.3–3.9)	0.018	3.1(1.2–8.3)
		=2	345(90.1%)	352(94.6%)	620(93.9%)	0.022	0.6(0.4–0.9)	0.459	1.2(0.7–2.2)
		>2	33(8.6%)	8(2.2%)	33(5.0%)	0.021	1.8(1.1–3.0)	0.012	0.4(0.2–0.8)
miR-143	Stage 1	<2	2(0.5%)	0(0.0%)	1(0.2%)	0.279	3.5(0.3–38.5)	—	—
		=2	375(98.4%)	368(99.2%)	647(98.0%)	0.647	1.3(0.5–3.3)	0.084	2.9(0.8–10.3)
		>2	4(1.0%)	3(0.8%)	12(1.8%)	0.332	0.6(0.2–1.8)	0.116	0.4(0.1–1.3)
miR-205	Stage 1	<2	5(1.3%)	0(0.0%)	3(0.5%)	0.13	2.9(0.7–12.2)	—	—
		=2	369(96.3%)	358(98.6%)	650(98.6%)	0.015	0.4(0.2–0.9)	0.988	1.0(0.3–3.0)
		>2	9(2.3%)	5(1.4%)	6(0.9%)	0.06	2.6(0.9–7.4)	0.489	1.5(0.5–5.0)
miR-9-3	Stage 1	<2	14(3.7%)	26(6.9%)	29(4.4%)	0.574	0.8(0.4–1.6)	0.084	1.6(0.9–2.8)
		=2	263(69.0%)	267(70.8%)	434(65.8%)	0.28	1.2(0.9–1.5)	0.094	1.3(1.0–1.7)
		>2	104(27.3%)	84(22.3%)	197(29.8)	0.382	0.9(0.7–1.2)	0.008	0.7(0.5–0.9)
miR-23a	Stage 1	<2	3(0.8%)	3(0.8%)	0(0.0%)	—	—	—	—
		=2	339(90.9%)	342(92.2%)	640(97.0%)	2.44 × 10^−5^	0.3(0.2–0.6)	0.003	0.4(0.2–0.8)
		>2	31(8.3%)	26(7.0%)	20(3.0%)	**2.00** × **10**^**−4**^	**2.9(1.6**–**5.2)**	0.011	2.2(1.2–4.0)
	Stage 2	<2	9(1.0%)		15(0.9%)	0.758	1.1(0.5–2.6)		
		=2	790(91.9%)		1559(95.6%)	1.30 × 10^−4^	0.5(0.4–0.7)		
		>2	61(7.1%)		57(3.5%)	**5.83** × **10**^**−5**^	**2.1(1.5**–**3.1)**		
	Combined	<2	12(1.0%)		15(0.7%)	0.301	1.5(0.7–3.2)		
		=2	1129(91.6%)		2199(96.0%)	4.79 × 10^−8^	0.5(0.3–0.6)		
		>2	92(7.5%)		77(3.4%)	**5.83** × **1**0^−8^	**2.3(1.7**–**3.2)**		
miR-146a	Stage 1	<2	5(1.3%)	0(0.0%)	3(0.5%)	0.129	2.9(0.7–12.2)		
		=2	350(91.4%)	361(99.4%)	656(99.4%)	1.57 × 10^−11^	0.1(0.02–0.2)	0.912	1.1(0.2–6.0)
		>2	28(7.3%)	2(0.6%)	1(0.2%)	**1.21** × **10**^**−11**^	**52.0(7.0**–**383.6)**	0.258	3.7(0.3–40.4)
	Stage 2	<2	59(6.9%)		77(4.8%)	0.032	1.5(1.0–2.1)		
		=2	679(79.4%)		1470(92.0%)	2.12 × 10^−19^	0.3(0.3–0.4)		
		>2	117(13.7%)		51(3.2%)	**1.08** × **10**^**−22**^	**4.8(3.4**–**6.8)**		
	Combined	<2	64(5.2%)		80(3.5%)	0.021	1.5(1.1–2.1)		
		=2	1029(83.1%)		2126(94.2%)	7.11 × 10^−26^	0.3(0.2–0.4)		
		>2	145(11.7%)		52(2.3%)	**8.43** × **10**^**−31**^	**5.6(4.1**–**7.8)**		
miR-301a	Stage 1	<2	19(5.0%)	11(2.9%)	9(1.4%)	**4.71** × **10**^**−4**^	**3.8(1.7**–**8.5)**	0.078	2.2(0.9–5.3)
		=2	352(92.9%)	357(95.2%)	645(97.7%)	1.32 × 10^−4^	0.3(0.2–0.6)	0.026	0.5(0.2–0.9)
		>2	8(2.1%)	7(1.9%)	6(0.9%)	0.106	2.4(0.8–6.8)	0.184	2.1(0.7–6.2)
	Stage 2	<2	41(4.8%)		30(1.9%)	**3.87** × **10**^**−5**^	**2.6(1.6**–**4.3)**		
		=2	792(93.2%)		1543(97.0%)	1.10 × 10^−5^	0.4(0.3–0.6)		
		>2	17(2.0%)		18(1.1%)	0.085	1.8(0.9–3.5)		
	Combined	<2	60(4.9%)		39(1.7%)	**9.23** × **1**0^−8^	**2.9(1.9**–**4.4)**		
		=2	1144(93.1%)		2188(97.2%)	8.77 × 10^−9^	0.4(0.3–0.5)		
		>2	25(2.0%)		24(1.1%)	0.021	1.9(1.1–3.4)		

Bonferroni correction for the number of CNVs tested by the conditional analysis, P value less than 0.05/21 = 0.0024 was supposed significant. Discrepancy between numbers of individuals is due to missing genotyping data.
